# A qualitative study of factors influencing male participation in fertility research

**DOI:** 10.1186/s12978-020-01046-y

**Published:** 2020-11-23

**Authors:** Alyssa F. Harlow, Amy Zheng, John Nordberg, Elizabeth E. Hatch, Sam Ransbotham, Lauren A. Wise

**Affiliations:** 1 Department of Epidemiology, Boston University School of Public Health, 715 Albany Street, Boston, MA 02188 USA; 2 Department of Information Systems, Boston College Carroll School of Management, Chestnut Hill, MA 02467 USA

**Keywords:** Male fertility, Qualitative research, Male participation, Reproductive health, Research participation, Preconception cohort

## Abstract

**Background:**

Although fertility is a couple-based outcome, fertility studies typically include far fewer males than females. We know little about which factors facilitate or inhibit male participation in fertility research. In this study we aimed to explore factors that influence male participation in fertility research among North American couples trying to conceive.

**Methods:**

We conducted a qualitative research study of male participation in Pregnancy Study Online (PRESTO), a prospective preconception cohort of couples actively trying to conceive in Canada and the United States. Between January–August 2019, we carried out 14 online one-on-one in-depth interviews and one online focus group of males and females with varying levels of participation. The in-depth interviews included females who enrolled in PRESTO but declined to invite their male partners to participate (n = 4), males who enrolled in PRESTO (n = 6), and males who declined to participate in PRESTO (n = 4). The focus group included 10 males who enrolled in PRESTO. We analyzed the transcriptions using inductive content analysis.

**Results:**

Male and female participants perceived that fertility is a women’s health issue and is a difficult topic for men to discuss. Men expressed fears of infertility tied to masculinity. However, men were motivated to participate in fertility research to support their partners, provide data that could help others, and to learn more about their own reproductive health.

**Conclusions:**

Male participation in fertility studies will improve our understanding of male factors contributing to fertility and reproductive health issues. Results indicate a need for more education and health communication on male fertility to normalize male participation in fertility and reproductive health research.

**Plain English Summary:**

Men are much less likely than women to participate in research on fertility and pregnancy. However, it is important for men to participate in fertility research so that we gain a better understanding of male factors that impact fertility and pregnancy outcomes. In this qualitative study, we interviewed men and women from Canada and the United States who were trying to become pregnant to understand why men choose to participate in fertility research, why men choose not to participate in fertility research, and why women choose not to invite their male partners to participate in fertility research. We found that both men and women believe fertility is a woman’s health issue. Men find it difficult to talk about pregnancy and fertility and have fears of infertility tied to masculinity. However, men are motivated to participate in fertility research to support their partners, to help others, and to learn more about their own reproductive health.

## Background

Although fertility is a couple-based outcome and male factors account for 50% of infertility cases [1], most fertility studies enroll females only and focus only on female determinants. Over the past decade, the National Institutes of Health and other funding bodies have placed greater emphasis on advancing scientific knowledge on male factors that influence fertility [2]. While advanced male age [3, 4], smoking [5–7], obesity [8–10], and other lifestyle factors [11–13] may affect fertility, other male factors are unknown. The lack of knowledge on male factors is partially due to a lack of available data, and the limitations of using female proxies for male exposures [14]. Few males participate in preconception and reproductive studies, and we know little about which factors facilitate or inhibit male participation in fertility research. Understanding these factors can inform targeted recruitment efforts and potentially improve male participation rates in fertility studies.

One barrier to male engagement in reproductive health research is that men may feel disconnected from fertility because women get pregnant, not men [15]. Cultural and gender norms surrounding pregnancy also contribute to men and women perceiving that pregnancy is a woman’s domain and that men are not expected to be engaged in the preconception process [15–18]. These cultural norms may also cause investigators not to invite men to participate in studies on reproductive health [19]. Clinical treatment of infertility (e.g., assisted reproduction) tends to focus on female bodies, which in turn contributes to an environment where men feel they are not equal partners in the reproductive process [19–21]. Most research on male engagement in fertility or pregnancy processes includes either couples experiencing infertility, or men from the general population who are not actively trying to conceive with a partner. Few studies examine male participation in fertility research among couples actively trying to conceive.

This study intends to understand the factors contributing to male participation in Pregnancy Study Online (PRESTO), a large preconception cohort study of North American pregnancy planners. Using qualitative research methods, we interviewed men and women from PRESTO to identify factors contributing to participation (or lack thereof) by men in fertility research.

## Methods

### Study design

We conducted a qualitative substudy among participants of PRESTO, an ongoing web-based prospective cohort study of North American couples attempting to conceive. The study design and methods of PRESTO were described previously [22]. PRESTO aims to identify factors associated with couple fecundability, measured by time to pregnancy. Eligible participants include women 21–45 years old who live in the United States or Canada, live with a male partner, and are actively trying to conceive without fertility treatments. Females complete a baseline questionnaire, after which investigators encourage them to invite their male partners to participate. Men who choose to participate complete a baseline questionnaire and an optional dietary questionnaire. Investigators ask a subset of men who completed the baseline questionnaire to provide a semen sample. About 57% of female participants invite their male partners to complete the baseline questionnaire, and 50% of those invited men agree to participate [22].

To understand factors contributing to male participation in PRESTO and fertility research in general, we conducted web-based one-on-one interviews. We interviewed men who were invited but chose not to participate in PRESTO, men who were invited and participated in PRESTO, and women who did not invite their male partners. We then conducted one web-based focus group with a separate sample of male PRESTO participants to confirm emerging results from the one-on-one in-depth interviews [23].

### Sampling scheme

To generate our sample, we used criterion-based purposeful sampling [24]. We selected participants based on characteristics (i.e., criteria) that allowed for detailed exploration of themes relevant to our research questions [24, 25]. Sample criteria included variables associated with male enrollment in a prior quantitative analysis of PRESTO data [22]. We then consulted the principal investigator of PRESTO, a subject-matter expert, to identity additional variables to include in our sampling scheme. We ranked criteria as primary criteria (i.e., variables given first priority in sample structure), secondary criteria (i.e., variables included in sample structure, but considered less important than primary criteria), and tertiary criteria (i.e., variables not specified in sample composition, but monitored during recruitment to promote sample diversity) [25].

Our only primary criterion was type of PRESTO respondent: male non-respondents (men who were invited to PRESTO and chose not to participate in any capacity), male respondents (men who were invited to PRESTO and agreed to participate in the baseline and dietary survey, and provided a semen sample), and female non-inviters (women who did not invite their male partners to participate). Our secondary criteria included education (< college vs. ≥ college education), geographical region (four US census regions plus Canada), and pregnancy attempt time at PRESTO baseline (< 3 vs. ≥ 3 cycle attempts). Tertiary criteria included female age (< 30 vs. ≥ 30 years), race/ethnicity (Non-Hispanic White, Non-Hispanic Black, Non-Hispanic other race, Hispanic), and relationship length (< 5 vs. ≥ 5 years).

For in-depth one-on-one interviews, we aimed to recruit 3–6 participants of each respondent type. Within each respondent type, we purposefully-sampled for diversity of our secondary and tertiary criteria. (Table 1 describes the purposeful sampling matrix) [24]. For our focus group, we aimed to recruit a separate sample of 5–10 men who did not participate in the one-on-one interviews and completed at least the PRESTO baseline questionnaire.

**Table 1:** Sampling Matrix for in-depth interviews

Secondary sample criteria	Primary sample criteria			
	Male respondent	Male non-respondent	Female non-inviters	Total
Education	≥ 1 with less than college education and ≥ 1 with college education	≥ 1 with less than college education and ≥ 1 with college education	≥ 1 with less than college education and ≥ 1 with college education	
US census regions and Canada	≥ 2 different regions represented	≥ 2 different regions represented	≥ 2 different regions represented	At least all five represented across total sample
Pregnancy attempt time at enrollment in PRESTO	≥ 1 with < 3 cycles and ≥ 1 with 3–6 cycles	1 with < 3 cycles and ≥ 1 with 3–6 cycles	1 with < 3 cycles and ≥ 1 with 3–6 cycles	
Targeted n	3–6	3–6	3–6	9–18

Across total sample, monitor for diversity in race/ethnicity, female partner age and relationship duration

Sample matrix developed using steps outlined in Ritchie, Lewis & Elam (2013)

### Inclusions and exclusion criteria

We excluded participants trying to conceive for more than 6 months at enrollment. This is an exclusion criterion for the analysis of fecundability data in PRESTO. We additionally reasoned that women who had been trying to conceive for a longer time might be less likely to invite their male partners. To ensure that participants were able to recall thought processes in choosing whether or not to participate in PRESTO (or in choosing not to invite their partners), participants were eligible for in-depth interviews if they received their invitation to participate in PRESTO (males) or completed the baseline questionnaire (females) within the previous 6 months. Due to a lower response rate, we relaxed this eligibility requirement to invitations within the previous 12 months for focus group participants.

### Participant recruitment

We conducted recruitment and data collection for the in-depth interviews before the focus group. We identified a list of eligible participants that we updated weekly. The Principal Investigator of PRESTO first reviewed the list of eligible participants and identified male respondents she felt would provide information-rich data based on their participation in PRESTO. “Information-rich” data provides in-depth and detailed information on the phenomenon of interest [25]. In the case of our study, we identified male respondents who were particularly engaged in PRESTO, as demonstrated by their e-mail correspondence and timeliness of survey completion. We contacted these information-rich participants first, followed by randomly selected participants from the remaining list of eligible participants. We e-mailed selected participants details of the qualitative study, including the research aims, types of questions, anticipated length of the interview, interview format, and compensation. We repeated this process for focus group participants after completion of in-depth interviews, with the focus group date established a priori. Participants received a reminder e-mail one week and 24-h before the scheduled interview or focus group.

### Study sample

We invited 26 male respondents, 51 male non-respondents, and 21 females to participate in the in-depth interviews (contacted no more than twice). The final sample for in-depth interviews included 6 male respondents (response percentage: 23%), 4 male non-respondents (8%), and 4 female non-inviters (19%). We contacted 170 men for the focus group. The final focus group sample included 10 male participants (6%).

### Data collection

We held the interviews and the focus group between January-August 2019 using Zoom Video Communications © (Zoom) web-conference video technology (version 4.1), consistent with PRESTO’s online data collection approach [22]. The video-chat method allowed for face-to-face contact with participants and reading of non-verbal and contextual data. Three participants (2 female non-inviters and 1 male non-respondent) opted for an audio-only interview.

A female PhD student trained in qualitative methods (A.H.) conducted in-depth interviews and moderated the online focus group. Interviews followed a semi-structured interview guide tailored to a participant’s specific respondent group. A researcher experienced in qualitative research methods (S.R) reviewed the interview guide for content, format and interpretability. Questions included participants’ experience, behaviors, opinions/values, and feelings about choosing to participate, choosing not to participate, or choosing not to invite one’s male partner. Interviews also asked participants their opinions of male participation in fertility research in general, and about specific barriers or facilitators to their participation. Preliminary analysis of the in-depth interviews informed the focus group interview guide. The interview guide followed a similar format as the in-depth interviews; however, the moderator additionally relayed findings from the in-depth interviews to triangulate preliminary thematic findings [25]. We digitally recorded interviews through Zoom software. Each participant received a $100 gift card.

### Ethical approval

The Boston University Medical Campus institutional review board approved this research.

### Analysis

We uploaded professional transcripts of the interviews to NVIVO Version 12. Three study staff members (1 Ph.D. level and 2 masters level coders) conducted an inductive content analysis of the interviews [26]. We analyzed all interviews of the same respondent type separately, beginning with male respondents. To understand the data as a whole, coders first read through all transcripts of the respondent type. Coders then began coding one information-rich interview by reading through the interview and summarizing important phrases, sections, or quotations. Using emergent patterns, coders rearranged the summarized sections through an iterative process based on how they relate to each other [27]. Coders labeled each arrangement to create a set of codes. The coders met throughout this process to discuss and review the proposed set of codes, and address inconsistencies or disagreements using a consensus approach [28]. Using an agreed-upon codebook, each coder then separately coded the remaining interviews and met periodically throughout the process to review and update codes and reach consensus on any discrepancies. We repeated this process for male non-respondent interviews, female non-inviter interviews, and the focus group. Coders used the same codebook for each respondent type, however the codebook evolved throughout the coding process. Throughout the coding process, coders reviewed the video of each interview to better immerse themselves in the data, and identify non-verbal cues. We analyzed the final coded interviews for patterns within and across respondent types to identify emergent factors important to male participation in fertility research.

## Results

Table 2 presents descriptive statistics of the purposeful sampling criteria. In each respondent type, there was variation in education, geography, pregnancy attempt times, female age, and relationship length. However, 85.7% of the one-on-one interview participants and 80% of the focus group participants were non-Hispanic white. The average length of the in-depth interviews was 37 min, and the focus group lasted 1 h and 23 min.

**Table 2:** Demographic characteristics of participants by respondent type^a^

	One-on-one interviews			Focus group participants
	Male respondents (n = 6)	Male non-respondents (n = 4)	Female non-inviters (n = 4)	Male respondents (n = 10)
	n (%)	n (%)	n (%)	n (%)
Race/ethnicity				
Non-hispanic white	6 (100)	2 (50)	4 (100)	8 (80)
Non-hispanic Black	0 (0)	0 (0)	0 (0)	0 (0)
Non-hispanic other race	0 (0)	2 (50)	0 (0)	0 (0)
Hispanic/Latina	0 (0)	0 (0)	0 (0)	2 (20)
Education				
< College	1 (17)	1 (25)	3 (75)	2 (20)
≥ College	5 (83)	3 (75)	1 (25)	8 (20)
Geography				
US Northeast	2 (33)	1 (25)	0 (0)	2 (20)
US South	1(17)	1 (25)	2 (50)	3 (30)
US Midwest	2 (33)	1 (25)	2 (50)	1 (10)
US West	1 (17)	1 (25)	0 (0)	2 (20)
Canada	0 (0)	0 (0)	0 (0)	2 (20)
Pregnancy attempt time at enrollment^b^				
< 3 cycles	5 (83)	3 (75)	3 (75)	7 (70)
≥ 3 cycles	1 (17)	1 (25)	1 (25)	3 (30)
Relationship length^c^				
< 5 years	1 (17)	1 (25)	2 (50)	2 (20)
≥ 5 years	5 (83)	3 (75)	2 (50)	8 (80)
Female age^d^				
< 30 years	1 (17)	2 (50)	4 (100)	8 (80)
≥ 30 years	5 (17)	2 (50)	0 (0)	2 (20)
Male age^e^				
< 30 years	1 (17)	1 (25)	4 (100)	3 (30)
≥ 30 years	5 (17)	3 (75)	0 (0)	7 (70)

^a^Male respondents are men who participated in Pregnancy Study Online (PRESTO); male non-respondents are men who were invited to PRESTO but declined to participate; female non-inviters are women who were in enrolled in PRESTO but declined to invite their male partners to participate

^b^Pregnancy attempt time at enrollment in PRESTO. Reported by female partners.

^c^Refers to relationship length at time of enrollment or invitation to PRESTO

^d^For male participants, refers to age of female partner at the time of enrollment or invitation to PRESTO

^e^For female participants, refers to age of male partner at the time of enrollment in PRESTO

Five factors emerged from the data important to male participation in PRESTO and/or fertility research in general (Table 3), including: (1) seeking knowledge but fearing results; (2) recognizing the importance of fertility issues; (3) believing fertility and pregnancy are women’s issues; (4) avoiding difficult conversations; and (5) supporting their partner.

**Table 3:** Emergent factors influencing male participation in fertility research and supporting quotes

Factors	Summary	Supporting quotes
Seeking knowledge but fearing results	Men were interested in participating in PRESTO to gain knowledge on reproductive health, however they expressed fear of receiving results indicating poor semen quality	“I was curious to see what type of results that I would get back.”—**Male Respondent** “But when like when there was the whole like sample collection thing, and you got numbers about like, I mean, like that’s cool. Like that’s valuable to me. Like that’s information that I got that I didn’t have before. And so that is motivating to me.”—**Male Respondent** “I think it’s very deep rooted, deep seeded, this belief of manliness and virility, and being able to conceive a child and you know, the inability to do so, to be seen as impotent, and you know, not effective, it, it’s sort of, I think it’s, it’s closely intertwined with notions of manliness and masculinity.”—**Male Non-Respondent** “I think it’s the same as like all these other things we’re talking about, like the fear of not being masculine. A lot of us have grown up in the world where if you can’t do something like that, then you’re not really a man, which, you know, obviously isn’t true, but, you know, that thought in society.”—**Focus Group Participant**
Recognizing the importance of fertility issues	Having knowledge on or a personal connection to fertility issues was positively associated with men participating in fertility research and women choosing to invite their male partners	“It’s just like he’s never been around infertility. I mean, his mother and his father didn’t have any problems conceiving. They have three children, so there’s never been anything, like, in his life that would make him think that having a child is a matter of timing, and a matter of patience, and a matter of, you know, anything else that you might need in order to make your body ready to have a child.”—**Female Non-Inviter ** “We’ve personally benefited from a lot of the research that goes into infertility, you know, learning about how other couples have managed, learning about the statistics and probability of getting pregnant, which helps us to go into it with a more, kind of realistic mindset so we kind of know what we’re getting into and know what to expect.”—**Male Respondent** “So, anyway, this being my first time having a child at all, I never had challenges with it before. But I had heard of others who had challenges and figured that that might you know, potentially happen to me.”—**Male Respondent **
Believing fertility and pregnancy are women’s issues	Men and women perceived fertility and pregnancy to be women’s issues. This was driven by several factors, including that women get pregnant and carry the baby, male fertility is rarely discussed in education or healthcare systems, and because of gender norms related to pregnancy	“So, I feel like he thinks that it’s a woman’s issue. It’s not that he’s not sympathetic and, you know, and wants do whatever he can, especially in our situation to help, but except for taking the survey, I guess. But, yeah, he just thinks it’s more on that end, like, okay, it’s something you have to deal with, and you have to do stuff, and you have to do this and I just, don’t have to worry about”—**Female Non-Inviter** “Yeah, and I think it was kind of girlie to him in a sense too, you know, because girls are the ones that are like, hey, let’s have a kid. And this is how you gotta do it.”—**Female Non-Inviter** “I’ll add the thought that sperm count is something that isn’t measured in any regular physical checkup normally for guys.”—**Focus Group Participant** “Just even in the education system throughout school, that if you look at – at least in Canada, the – the sex education system, there was a lot of focus on, I guess, if you are going to create a baby, , these are the things that could go wrong, but a lot of that focus wasn’t directed directly at potential problems on the male side, at least from my experience.”—**Focus Group Participant** “I think that the literature is more geared towards women. And I think that is a very broad statement, it applies to kind of academic research, and, you know, things of that nature. But it also applies to kind of more, you know, consumptive literature, you know, guides on how, how to get pregnant, how to be pregnant, how to be a mom. I think there’s more literature, you know, addressing the women, or the mom’s, kind of point of view than kind of necessarily the fathers. And so, I think for men to participate in this research helps to expand that body of literature to be able to address men more comprehensively. And I think just from a purely scientific basis, it’s probably needed, as well.”—**Male Non-Respondent** “Pregnancy is tremendously impactful to all people involved. But especially to women, because it literally changes their bodies and, um, and they sacrifice so much to be able to produce a kid.”—**Male Respondent**
Avoiding difficult conversations	Pregnancy and fertility were difficult topics for men to discuss, particularly with other men	“I don’t have a single friend that I talk to about this stuff. And so, it feels like a little bit more of like a guarded topic, even though it really should – and it’s just for whatever reason it does feel that way”—**Male Respondent** “I guess the global theme has been it might be a bit taboo for men to talk about it, to discuss it, to research, to look into it. I think it’s more seen as that’s something women talk about. Men shouldn’t discuss that amongst themselves.”—**Focus Group Participant** “Besides, like I said, the friends probably thinking it silly if he mentioned it and, you know, guys, like, when they talk about stuff, and they might laugh…. Then, you know, they like to sound masculine and cool. So, of course, he wouldn't want to tell them, or at least, wouldn't want to be doing it where they would find out.”—**Female Non-Inviter **
Supporting their partner	A strong motivation for participation in fertility research was to support the female partner. However women were hesitant to invite their partners because they did not want to “push” them to participate	“I know that it was something that I, by doing it for her, would help her out, in that journey. Both of us want to have kids, obviously. So, it was just something that I knew that she wanted me to do. So, I did it for her.”—**Focus Group Participant** “I mean like if – I suppose if my wife had talked to me about it before I got the email, then that absolutely would have put it on my radar and I would have been watching for that email… I suppose just – I think having my wife talk to me about it before the email would have really helped.”—**Male Non-Respondent** “Um, probably if I, you know, was like, “Oh, look,” and just told him how important it is. Like– he’s pretty good with that kinda stuff. Like he’s usually willing. It’s just honestly, it was probably more of my own thoughts that I was like, “Oh, he’s busy, you know.” I don’t know if he would have said no or not. I don’t think he would have said – usually, if I ask him to do things, he’ll do it.”—**Female Non-Inviter**

### Seeking knowledge but fearing results

Male respondents participated to gain an understanding of their own reproductive health, particularly knowledge of semen quality or dietary behaviors. Males invited by PRESTO investigators to provide semen samples commented on how getting concrete results back about their reproductive health was a strong motivation for participation, and an opportunity for empowerment.

*“And just like, knowledge is power. I was looking forward to getting a little bit more knowledge about my own body.”*

For male respondents who struggled to conceive in the past, gaining knowledge about their reproductive health was an important benefit of the study. Research participation was one extra thing they could do to help their fertility.

*“…the value about sperm count and motility I think is really interesting because so, we’ve – we have had some issues getting a pregnancy to stick. And like, that’s another piece of data that’s valuable in helping that. And so, we’re still working through our fertility situation. And the opportunity to have maybe a little bit of data or understanding of what’s going on through this, it’s another opportunity.”*

While knowledge motivated participation, it was a barrier as well. Male respondents and focus group participants feared receiving information that their semen is of poor quality. Participants felt that some men might prefer to have no information on their reproductive health rather than bad information.

*“People are more happy to know that they are virile men and they don’t want to have like questioning of what they have wrong with them. And so,– that’s my assumption, that people would rather not know or go under the idea that they have nothing wrong versus going through this process.”*

Men who participated in the semen testing activities of PRESTO confirmed their nervousness around getting results back, and subsequent relief upon getting a good result.

*“I did the semen sample already. I’m one of the lucky ones. Everything came back in the optimal range and – I’ve got to tell you, I high-fived my wife. I was like so excited. I was like, “Yeah, yeah, I got it,” because it was definitely a level of concern that existed in our minds.”*

Interviewees connected these fears of obtaining bad results to feelings of vulnerability, and to the idea that virility is intertwined with the cultural concept of masculinity. Both respondents and non-respondents discussed how receiving a result that indicates a fertility problem might compromise their own feelings of masculinity.

*“You get a result that’s not outstanding and you can feel like less of a man.”*

### Recognizing the importance of fertility issues

Recognition that fertility, and the process of conceiving, is an important and potentially emotionally taxing health issue was a factor in male participation or lack of participation in PRESTO, and for women in their decision to invite their partners. Many women felt their male partners had little knowledge about fertility or preconception. Some perceived that their partners believed that pregnancy is always simple, quick, and easy rather than a sometimes complex, lengthy, and stressful process. These women felt their partner’s beliefs stemmed from little exposure to experiences of infertility, either personally or in their families.

*“I just think he doesn’t understand, you know. Like he thinks it’s easy. It’s like one and done. And yeah, okay, it takes a few months”*

These feelings contributed to some women’s decisions not to invite their partners to participate. Because they perceived their partners lacked knowledge, some women believed their partners would not invest in the research process.

*“I don’t think he would’ve been invested in it because he really had no reason to think that there would ever be a problem in conceiving a child.”*

One male non-respondent discussed how he personally had never been exposed to experiences of infertility. He expressed doubt that lifestyle factors play a role in conception because he had impregnated his partner while using marijuana and other drugs. His own experience with successful conception led him to believe that fertility research offered little benefit to him or society.

*“I didn’t live a healthy lifestyle. I mean, I did exercise and stuff here and there, but I mean, I smoked. I smoked pot all the time. I mean, I grew up smoking pot. And another thing is, at the time she got pregnant, I was on narcotics for my neck as well. Now they say if you – take this, that, it'll lessen your chances and all that. It's not true, you know.”*

In contrast, personal experiences with pregnancy loss positively influenced participation. Some mentioned personally benefitting from fertility and pregnancy research, which encouraged their own participation in PRESTO. One non-respondent male who expressed regret about not participating in PRESTO revealed that a prior miscarriage increased his desire for more information on his personal reproductive health, but also the desire to help others going through similar scenarios.

*“Oh, yeah, so, we had a miscarriage last year, and so, because of that, I think I would have been much more likely to want to participate, understanding that, you know, because of that negative experience, we sort of wanted to get more information, and so we’d understand the value of helping to provide that information to researchers. So, you know, again, another answer that kind of suggests that I would have done the opposite of what I did.”*

Others mentioned that having friends who experienced fertility issues helped them realize that they might have their own difficulties conceiving. Participants felt this knowledge increased their desire to participate in order to help others.

*“I’m sure we all have friends who have tried unsuccessfully to have kids, and so just the idea that maybe if I can be one more data point that would help a study, you know, who knows, then maybe [it will] help more people who want to become pregnant – then I want to help out with that.*

### Believing fertility and pregnancy are women’s issues

Both women and men perceived that fertility and pregnancy are women’s health issues. Participants from each respondent type framed women as caring more about fertility and pregnancy than men.

*“If I had to say one thing though, it’s just that I suppose I feel like males in general are just not as interested in fertility stuff, as you guys are probably finding out.”*

Participants discussed how society often views subfertility or infertility as health issues that only affect women, and that researchers could improve recruitment of men in fertility studies by communicating that men can also contribute to couple subfertility or infertility.

*“I think there’s a perception – I think many people would agree with this –that infertility is a woman’s problem… I think in terms of getting more men to participate, if you can quantify when it is the male problem, if you can make a stronger case there and then the wife or the partner can then use that as a way to persuade hopefully their husband or spouse.”*

Cultural factors influenced beliefs that fertility and pregnancy are women’s issues. Several participants referenced fertility and pregnancy as feminine topics. These participants discussed traditional gender roles or cultural expectations for women to want children. One female participant mentioned how women are often the person in a relationship to initiate the process of trying to conceive. A male non-respondent mentioned how he viewed fertility and pregnancy as a “girl topic” because he rarely discussed pregnancy with his peers growing up, but perceived that women have these conversations often.

*“I haven’t had a lot of conversations growing up about like, “Oh how many kids do you want to have?” And, “What do you want to name your children?” And I never had conversations like that with my friends. So, I guess that’s just not a topic that for some reason I never went to with friends or anything growing up. But it was something that I, you know, would hear girls that I knew talking about a lot. And they would be interested in it, so I guess it just in my mind got associated with that’s a girl topic.”*

Some participants mentioned that male fertility is rarely discussed within educational and healthcare systems. Participants felt that men have to actively seek out information on fertility if they are interested in learning more. In contrast, they believed that the medical profession regularly discusses the topics of fertility and pregnancy with women.

*“And also, just like women have doctors’ appointments and stuff where they talk about fertility and things. I feel like men are often missing out on that and they don’t really have a chance unless they seek it out to discuss fertility.”*

In general, both respondents and non-respondents felt that most pregnancy-related literature, including books, online forums, and academic research focuses on the female perspective.

*“If you go to a forum or something that deals with trying to conceive or pregnancies, the balance, the gender balance is skewed heavily towards women, as opposed to men.”*

One non-respondent mentioned that expanding this consumptive literature might be one benefit of men participating in fertility research.

*“I think for men to participate in this research helps to expand that body of literature to be able to address men more comprehensively.”*

Both men and women mentioned that the perception of fertility and pregnancy being women’s issues also likely stems from the fact that biologically, women get pregnant and carry the child. The drastic changes to the female’s body would naturally lead women to greater interest and investment in the topic.

*“I suppose one of the reasons could be, during a pregnancy, there’s no changes to a man’s body, but the woman’s body goes through a lot. I suppose if there was a situation where there was going to be drastic changes to my body, I want to know about it too.”*

One non-respondent did not want overstep his boundaries, or take ownership of a process that primarily concerned female bodies.

*“One barrier that I feel a lot as a guy, me personally, and I think a lot of other guys, especially my age, feel, when it comes to this kinda thing, I think I kind of alluded to this a little bit, but like, not wanting to overstep my bounds, you know, like, not wanting to say to my wife, "Look, I've been doing a lot of research, and you know, this is what I think you need to start doing with your body," I think that – I feel that's really out of place, you know?”.*

Despite a perception of fertility and pregnancy as women’s issues, participants also felt that men should participate in fertility and pregnancy research to include the male perspective in this predominately female-dominated space.

*“I think that it's important for men to have their point of view represented.”*

### Avoiding difficult conversations

All participants felt that men struggled to talk about fertility and pregnancy, particularly among friends or peers.

*“I would never talk about this study to other guys that I know.”*

Most men perceived that women have an easier time talking about their experiences with pregnancy or trying to conceive.

*“I think it’s a lot easier for women to talk about it… than it is for a guy because I can’t walk up to my buddy be like, “Hey, man, you know, my sperm count’s low.” It’s just not kind of the conversation that you’d have. But, the conversations between my wife and her friends, I know always seem to lead to how much she wants to be a mom.”*

Participants felt that friends or peers might ridicule men for discussing pregnancy or fertility. One female participant discussed how her husband’s friends would think discussing fertility and pregnancy was a “silly” conversation, and that they would laugh at her husband if he brought it up.

*“…friends probably think it’s silly if he mentioned it and, you know, guys, like, when they talk about stuff, and they might laugh…”*

One focus group participant had openly discussed his participation in the study with his co-workers, and the participant perceived that it was an uncomfortable and “off-putting” topic.

*“I know for myself, working in the marine industry, where you do deal with that A-type masculine personality a lot, I’m an anomaly within my company. I openly talked about that I was in the [study]. But definitely, you could tell it was like an off-putting – like, it was a weird thing to talk about.”*

Participants relayed that one reason men feel uncomfortable talking about pregnancy and fertility is that it is a sensitive topic alluding to sex.

*“I was just gonna say that we can use these words of trying and conceiving, but when it comes down to it, I mean, you’re talking about sex and that just generally is not just something guys talk about in any sort of detail with each other.”*

One male non-respondent attributed his own discomfort to rarely discussing sex or reproduction with his own family growing up.

*“I think it's a cultural thing mostly. Um, I know my family, we grew up, you know, when I was a kid, we didn’t talk about things like sex or reproduction or, you know, women – women's health issues, stuff like that, it was just kind of impolite, and really, it was like considered impolite. You know, that's rude. You don’t talk about that stuff. I think a lot of that is still carried over into the modern age. I think that slowly the attitudes are changing, but, you know, I know a lot of people my age that are really uncomfortable talking about sex because that's the environment they grew up in.”*

Another focus group participant’s reservations around talking about pregnancy with peers were because it involved his wife’s body—a subject that he felt was hers to control rather than his.

*“Even if she’s not there, if it’s a group that involves her, if I’m talking about fertility and, uh, getting pregnant, her getting pregnant, I’m talking about her getting pregnant, not me. So I definitely don’t want to talk about it to try and give it – it’s her subject to control who knows about it.”*

### Supporting their partner

Nearly all male respondents cited their partner as the sole or primary reason for participation in PRESTO. Participating was a way to support their partner.

*“What motivated me was: Number one, I mostly did it to support my wife.”*

Beyond just agreeing to participate, partners often influenced participants to complete the study. Some participants completed the study activities so that that the data on them as a couple was as comprehensive as possible. Others felt the study would be more appealing to men if researchers invited couples to participate together. Completing the study together could create a teamwork mentality to motivate both participants to finish providing data.

*“If you’re on a team with your spouse, you not only make a commitment to the study, but to your spouse as well. Obviously, you’ll have more initiative to complete the questionnaires and participate in the focus groups.”*

Even among non-respondents, several emphasized that if their partners had reminded them about the study or encouraged them to participate, they likely would have participated.

*“Yeah, I mean, listen, if she told me to do it, I would just do it. If I saw that she was excited about it, then I would be much more inclined to do it, as well. So I think it’s as simple as that.”*

Similarly, women concurred that if they had invited their partners and talked to them about participating, then their partners would participate to support them.

*“I think if I would have talked to him about it as well as send the email, then probably. I mean, if I had just sent the email, he’d be like what is this? But if I talked to him at home, too… because I’m doing it and I’m interested and I’m excited or something, more like, he’d do it for me, you know.”*

However, women also did not want to feel as if they were pushing their partners to participate.

*“…but it was just one of those things that I didn’t wanna be pushing something on him that he didn’t necessarily feel was needed at this point.”*

## Discussion

This qualitative study on male participation in fertility research reveals several important factors contributing to male participation in a large preconception cohort study. All three respondent types—men who participated, men who chose not to participate, and women who chose not to invite their male partners to participate—held some common perspectives. Most participants perceived that fertility is a women’s health issue and is a difficult topic for men to discuss, and men expressed fears of infertility tied to masculinity. However, men participated in fertility research to support their partners, to provide data that could help others, and to learn more about their own reproductive health.

Both male and female participants felt that males were not connected to fertility and pregnancy in general, and that these were women’s health issues. They attributed this disconnect to a range of factors, including biology, societal barriers, and cultural norms. In prior research, men from the general United Kingdom population felt that women play a more important role in reproductive health decisions because they get pregnant and carry the child, and that society rarely encourages men to engage in discussions of fertility and pregnancy [15]. Our work demonstrates this belief also influences male engagement in fertility research among North American men and women actively trying to get pregnant. However, while men perceived fertility and pregnancy to be women’s issues, they also expressed a desire for more research and literature on male fertility, and felt that participating in PRESTO was an opportunity for the male voice to be heard. Researchers developing recruitment materials for male fertility studies may wish to emphasize that participation is an opportunity to provide the male perspective to fertility research.

Men desired to learn more about their own reproductive health but were apprehensive about results that might indicate fertility issues. Some men felt that fertility issues would make them feel less masculine, and that a fear of poor semen quality made them reluctant to participate in the semen testing substudy. These findings align with prior studies showing a perceived tie between virility and feelings of masculinity [29–31], and an association between diagnosis and treatment of infertility and poor mental health among men [32]. Tailoring counseling to men’s fertility results can help improve psychological adaptation to fertility issues [33, 34]. Therefore, one solution for researchers aiming to recruit men into fertility studies may be to offer resources on how to interpret any feedback they do receive, or resources for coping with difficulties conceiving.

Personal connections or past exposure to fertility issues positively contributed to men’s decision to participate or women’s decision to invite their male partners. Lack of knowledge on potential difficulties in the conception process may inhibit male participation in fertility research. A prior systematic review similarly found men tend to overestimate chances of spontaneous conception [35]. Many men in our study also expressed a general discomfort in discussing fertility issues, as reported in prior research [15]. Participants rarely talked about fertility or pregnancy with their peers, and felt that it was a sensitive topic because it alludes to sexual activity, or involves their partner’s body. These data highlight the importance of communicating research on fertility to the general public. Fertility researchers could work with health communication professionals to communicate study results to the public, or help to develop educational curriculums. If society normalizes talking about male fertility and male involvement in pregnancy, this may help men feel more comfortable discussing fertility and pregnancy with each other and with researchers.

Most men discussed participating as a way to support their female partners. Even men who had chosen not to participate conceded that they would have participated if their partners had encouraged them. The women’s perspective clearly demonstrated this as well; women reported that if they asked their partners to participate and conveyed that it was personally important to them, they were sure their partners would have participated. However, some women chose not to invite their partners partly because they did not want to feel like they were pushing them to do something against their will. This perception that participation in fertility research is a favor to the female partner likely decreases the likelihood of inviting male partners. These results indicate a potential tradeoff for researchers aiming to recruit male participants by using the female partners as gatekeepers. While the influence from the female partner likely facilitates male engagement, it simultaneously acts as a barrier for women to invite their partners to participate in the first place.

There are several limitations to this study. We attempted to recruit participants diverse in several socio-demographic factors. While we achieved diversity in education, geography, and pregnancy attempt times, 85.7% of the participants in the one-on-one interviews and 80% of the participants in the focus group were non-Hispanic White. In addition, we purposefully targeted certain men as information-rich cases that could provide insight on full research participation, but they were likely more engaged in fertility research than the average male participant. There were also low response rates among eligible participants. However, focus group participants confirmed the results derived from respondent and non-respondent interviews, which lends confidence to the trustworthiness of the data [25]. Finally, the interviewer for this study was a female Ph.D. student. A male interviewer might have made participants more comfortable to discuss the sensitive nature of male fertility and male participation in fertility research. In addition, there is the possibility of social desirability bias (i.e., participants providing responses they perceive to be socially acceptable). However, the experience that that female interviewer has in qualitative methods may have mitigated this. Finally, results may not be transferrable to other populations with different cultural norms and attitudes about sex or fertility.

## Conclusions

The recruitment of men into fertility studies will improve our understanding of male factors contributing to fertility and reproductive health issues. In this qualitative study, we reveal several factors affecting whether men participate in fertility research, as well as factors influencing a woman’s decision to invite her male partner to participate. Results suggest that more education and health communication on male fertility and reproductive health could help increase male participation in reproductive health research.

## Data Availability

Data sharing is not applicable to this article as no datasets were generated or analysed during the current study.
